# Diffusion-/perfusion-weighted imaging fusion to automatically identify stroke within 4.5 h

**DOI:** 10.1007/s00330-024-10619-5

**Published:** 2024-03-15

**Authors:** Liang Jiang, Jiarui Sun, Yajing Wang, Haodi Yang, Yu-Chen Chen, Mingyang Peng, Hong Zhang, Yang Chen, Xindao Yin

**Affiliations:** 1 Department of Radiology, Nanjing First Hospital, Nanjing Medical University, Nanjing, 210006 China; 2 Laboratory of Image Science and Technology, School of Computer Science and Engineering, Southeast University, Nanjing, 210096 China; 3 Department of Radiology, Affiliated Jiangning Hospital of Nanjing Medical University, Nanjing, 210000 China

**Keywords:** Stroke, Diffusion-weighted imaging, Perfusion-weighted imaging, Segmentation and classification, Onset time

## Abstract

**Objectives:**

We aimed to develop machine learning (ML) models based on diffusion- and perfusion-weighted imaging fusion (DP fusion) for identifying stroke within 4.5 h, to compare them with DWI- and/or PWI-based ML models, and to construct an automatic segmentation-classification model and compare with manual labeling methods.

**Methods:**

ML models were developed from multimodal MRI datasets of acute stroke patients within 24 h of clear symptom onset from two centers. The processes included manual segmentation, registration, DP fusion, feature extraction, and model establishment (logistic regression (LR) and support vector machine (SVM)). A segmentation-classification model (X-Net) was proposed for automatically identifying stroke within 4.5 h. The area under the receiver operating characteristic curve (AUC), sensitivity, Dice coefficients, decision curve analysis, and calibration curves were used to evaluate model performance.

**Results:**

A total of 418 patients (≤ 4.5 h: 214; > 4.5 h: 204) were evaluated. The DP fusion model achieved the highest AUC in identifying the onset time in the training (LR: 0.95; SVM: 0.92) and test sets (LR: 0.91; SVM: 0.90). The DP fusion-LR model displayed consistent positive and greater net benefits than other models across a broad range of risk thresholds. The calibration curve demonstrated the good calibration of the DP fusion-LR model (average absolute error: 0.049). The X-Net model obtained the highest Dice coefficients (DWI: 0.81; Tmax: 0.83) and achieved similar performance to manual labeling (AUC: 0.84).

**Conclusions:**

The automatic segmentation-classification models based on DWI and PWI fusion images had high performance in identifying stroke within 4.5 h.

**Clinical relevance statement:**

Perfusion-weighted imaging (PWI) fusion images had high performance in identifying stroke within 4.5 h. The automatic segmentation-classification models based on DWI and PWI fusion images could provide clinicians with decision-making guidance for acute stroke patients with unknown onset time.

**Key Points:**

• *The diffusion/perfusion-weighted imaging fusion model had the best performance in identifying stroke within 4.5 h.*

• *The X-Net model had the highest Dice and achieved performance close to manual labeling in segmenting lesions of acute stroke.*

• *The automatic segmentation-classification model based on DP fusion images performed well in identifying stroke within 4.5 h.*

**Supplementary Information:**

The online version contains supplementary material available at 10.1007/s00330-024-10619-5.

## Introduction

Intravenous (IV) tissue plasminogen activator (tPA), the dominant thrombolytic treatment for acute stroke, is recommended up to 4.5 h after symptom onset [1]. However, 14 to 27% of patients with stroke cannot receive IV tPA because of an unknown onset time (e.g., wake-up strokes or unwitnessed strokes) [2–4], leading to a relatively poor prognosis [5].

To address this problem, multimodal magnetic resonance imaging (MRI) technologies and CT have been used to identify stroke within 4.5 h [6–9]. Diffusion-weighted imaging (DWI)-fluid-attenuated inversion recovery (FLAIR) mismatch may be used to identify the onset time due to the immediate appearance of high-intensity signals on DWI, in contrast to the 1–4 h required on FLAIR imaging [9]. However, this mismatch concept achieves a specificity of only 0.60 to 0.80 with a moderate sensitivity of 0.5 to 0.6 [10]. Machine learning (ML), a scientific discipline that focuses on how computers learn from data, has been successfully applied to clinical datasets for developing robust risk models and redefining patient classes [11]. Recent studies have demonstrated that ML based on DWI and FLAIR images can outperform human readings in identifying stroke patients within 4.5 h [12, 13]. Our latest studies showed that the sensitivity of ML based on DWI and FLAIR images in identifying stroke onset time can reach 0.864, with a specificity of 0.845 [14]. However, some patients who may benefit from thrombolysis remain excluded. Approximately 80% of acute stroke patients have ischemic penumbra (perfusion-weighted imaging (PWI)-DWI mismatch) within 3 h of onset [15], and thrombolysis guided by PWI-DWI mismatch may be safe and associated with better outcomes [16]. Ho et al [13] identified new imaging features from PWI and showed that these features could be used to classify stroke onset time using ML, achieving a sensitivity of 0.788. The limited prior work on this topic may be due to a lack of key features or the difficulty of correctly establishing feature relationships based on a combination of the radiomics features from different imaging sequences. Deep learning, a subfield of ML, has exceeded the capabilities of classical statistical ML techniques for specific imaging tasks such as multiclass classification [17]. Normalization techniques are effective components in deep learning; examples include switchable normalization (SN), which learns to select different normalizers for different normalization layers of a deep neural network and has been proven to solve this problem [18]. This technique can be applied to DWI and PWI to obtain diffusion/perfusion-weighted imaging fusion (DP fusion) images, which contain all imaging features from DWI and time-to-maximum (Tmax) images. DP fusion images may be more accurate in identifying stroke onset time, but studies assessing their effectiveness in this regard are lacking.

The goal of this study was to develop ML models based on DP fusion images to further improve the classification performance in identifying stroke within 4.5 h, particularly in comparison with other ML models based on DWI and/or PWI. In addition, an automatic segmentation-classification model using deep learning was proposed to reduce the workload of radiologists and was compared with manual labeling methods. We hypothesized that the DP fusion model would achieve the best performance in determining the timing of acute stroke and that the automatic segmentation-classification model would achieve performance similar to that of manual labeling methods.

## Methods

### Study population

**Fig. 1:**
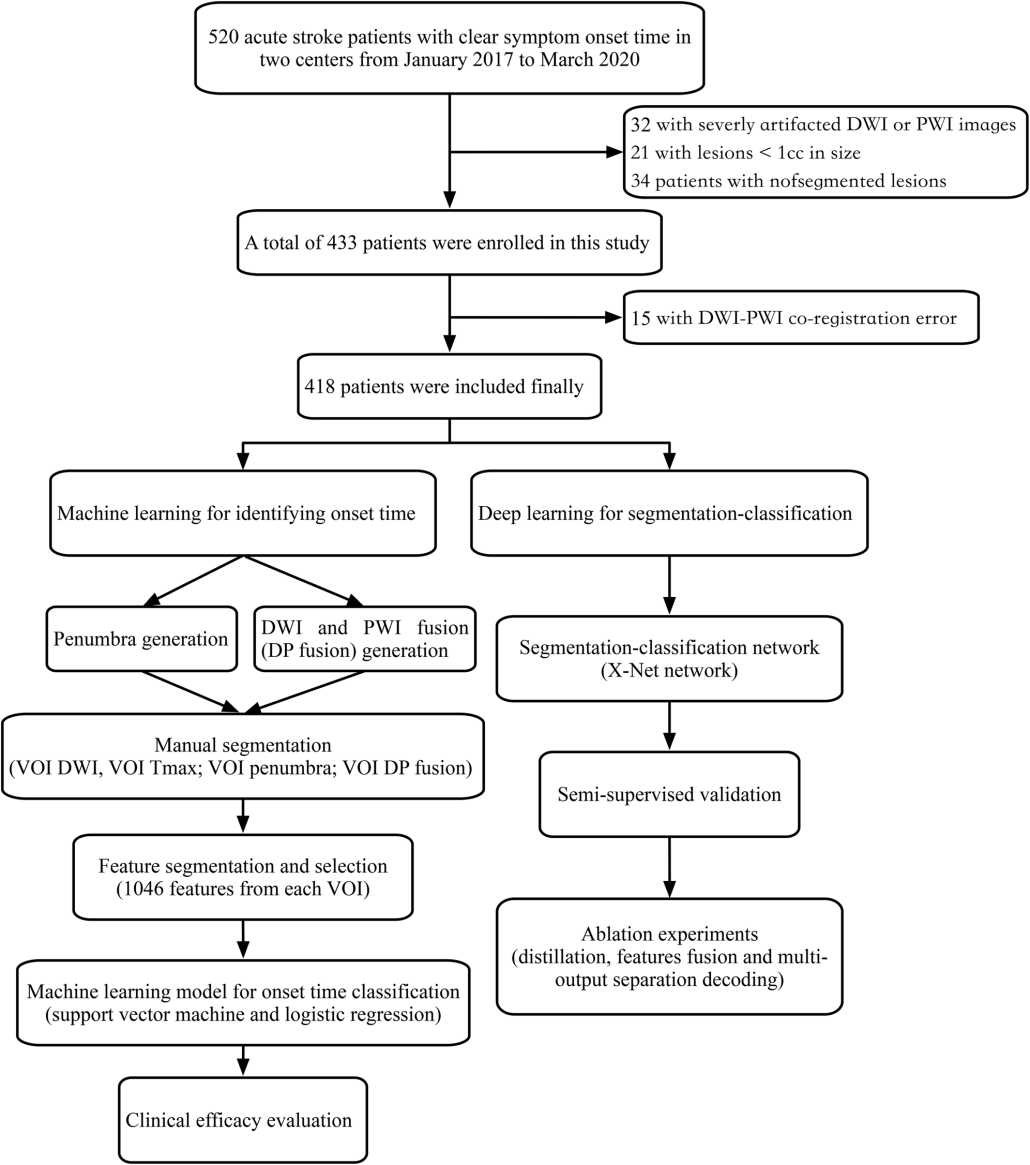
Flowchart of the included studies. DWI = diffusion-weighted imaging, DP fusion = diffusion/perfusion-weighted imaging fusion, PWI = perfusion-weighted imaging, VOI = volume of interest

Data from Nanjing First Hospital and the Affiliated Jiangning Hospital of Nanjing Medical University from January 2017 to December 2020 were retrospectively included. Anterior circulation acute ischemic stroke patients (AIS) were included if their symptom onset time was clear and within 24 h and if they underwent MRI scans, including both DWI and PWI sequences. Of 520 patients considered candidates for analysis, 32 patients with severely artifacts DWI or PWI images and 55 patients with lesions < 1 cc in size were excluded. Finally, a total of 433 patients were included for analysis. The flowchart shown in Fig. 1 outlines the patient selection process. According to the Trial of ORG 10172 in Acute Stroke Treatment (TOAST) classification system [19], AIS was classified into the following five subgroups: (1) large artery atherosclerosis (LAA); (2) small-artery occlusion (SAO); (3) cardioembolism (CE); (4) other determined cause (OC); and (5) undetermined cause (UND). The hospital review board of Nanjing Medical University approved the study protocol. All patients in this study provided written informed consent before the MRI examination. The patients were divided into two classes according to the onset time: positive (≤ 4.5 h) and negative (> 4.5 h).

### MRI protocol and processing

Patients in the two centers were scanned with the same MRI scanner and parameters. MRI scans were performed on a 3.0-T MRI scanner (Ingenia, Philips Healthcare) with an 8-channel receiver array head coil. A detailed description of the MRI protocol is provided in the Supplemental Materials (online). The PWI data were analyzed using RAPID software (IschemaView 5.0.2) to obtain Tmax images. The PWI processing component consists of motion correction and adjustments for different acquisition times in multislice echo-planar imaging scans, conversion of measured MR signals to estimated changes in transverse relaxivity, automatic detection of arterial input function and venous output, correction for the nonlinear effects of gadolinium tracers in bulk blood and capillaries, deconvolution, and final computation of perfusion parameters [20]. The Tmax parameter is a bolus-shape-independent estimate of time delay for blood delivery between a main feeding artery (e.g., middle cerebral artery) and tissue at a given spatial location, and the hypoperfusion is identified based on Tmax prolongation beyond a pre-specified threshold.

### Manual segmentation

All DWI and Tmax images of acute stroke patients were derived from DICOM format and converted to NII format using MRIcron software (https://www.nitrc.org/projects/mricron). The high-intensity signal infarction area on DWI (apparent diffusion coefficient < 600 × 10^–6^ mm^2^/s) and abnormal perfusion areas (Tmax > 6 s) on Tmax images were manually drawn as volumes of interest (VOIs) [20] using ITK-SNAP software (http://www.itksnap.org/pmwiki/pmwiki.php). Two certified neuroradiologists determined the VOI in consensus (Y-C.C., 8 years of experience, and M-Y.P., 15 years of experience).

### Coregistration and penumbra VOI generation

The DWI matrix size was 256 × 256 × 18, and the Tmax matrix size was 128 × 128 × 25. The ischemic penumbra cannot be obtained directly by subtracting the VOI of lesions in images of different scales and layers. Therefore, an image registration method was adopted in combination with affine transformation, and the mutual information was used as the optimization criterion. Advanced normalization tools (ANTs) were used to register DWI and Tmax sequences. After registration, the DWI images were essentially consistent with the Tmax images in terms of spatial position, and the image matrix size was changed from 256 × 256 × 18 to 128 × 128 × 25. The penumbra VOI was calculated using the following formula:

$$VOI\left(penumbra\right)=VOI\left(Tmax>6s\right)-VOI\left(DWI\right),$$

Of the 433 patients, 15 patients were further excluded because of coregistration error.

### DP fusion

The DP fusion image was obtained by fusing the DWI and Tmax images after registration. The image fusion method is shown in the following formula:

$${I}_{Fuse}=\frac{{I}_{dwi}-{\mu }_{dwi}}{{\sigma }_{dwi}}+\frac{{I}_{tmax}-{\mu }_{tmax}}{{\sigma }_{tmax}},$$

Where I_dwi_ and I_tmax_ are the DWI and Tmax images after registration, I_Fuse_ is the fused image, and μ and σ are the mean and variance of the image, respectively. The DP fusion images contain all imaging features of the DWI and Tmax images.

### Imaging feature extraction and selection

The radiomic features of the VOI_DWI_, VOI_Tmax_, VOI_penumbra_, and VOI_fusion_ were computed using PyRadiomics software (version: 3.0.1, https://pyradiomics.readthedocs.io/en/latest/), which follows the image biomarker standardization initiative (IBSI). The radiomics features covered six categories: shape-based (3D) features; first-order statistical features; gray-level cooccurrence matrix (GLCM); gray-level run-length matrix (GLRLM); gray-level size-zone matrix (GLSLM); gray-level dependence matrix (GLDM). Finally, a total of 1046 features were extracted from each VOI. The mean and standard deviation of features were normalized using the Z-score method. To filter redundant features and reduce feature dimensions, the t test was first used to identify features that could significantly differentiate between the onset time groups (*p* < 0.05). Then, the least absolute shrinkage and selection operator (LASSO) method with tenfold cross-validation, a suitable method for high-dimensional data regression, was used to select the most useful predictive features.

### Machine learning model

Two common ML algorithms were used to develop the classifier models: a support vector machine (SVM) [21] and logistic regression (LR) [22]. All training processes were performed in R software with the caret package. The models were evaluated using fivefold cross-validation. The 418 patients remaining after the previous exclusion process were divided into training and test sets at a ratio of 4:1. That is, 335 patients in each fold were included in the training set, and 83 patients were included in the test set.

### Deep learning for lesion segmentation and classification

**Fig. 2:**
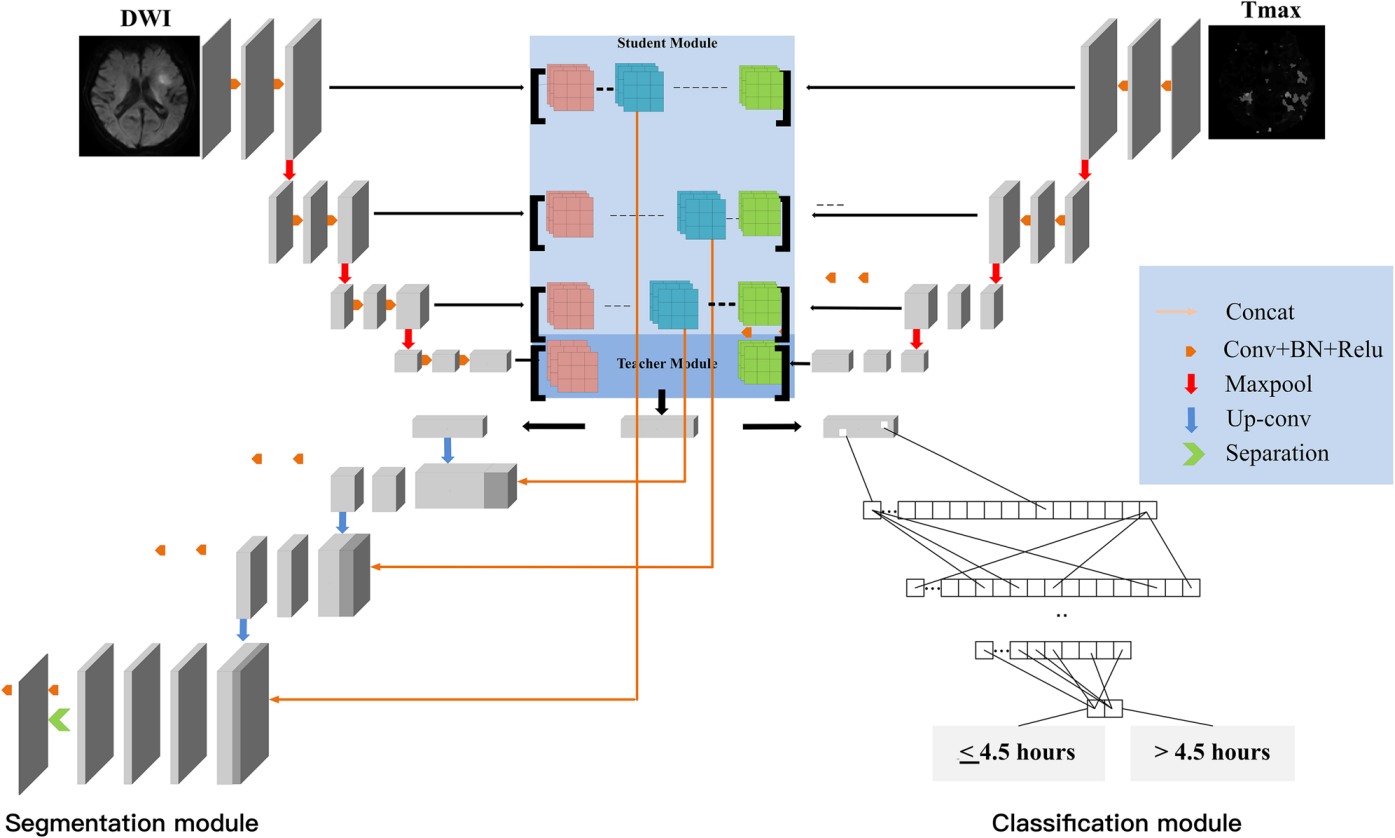
Schematic of the X-Net architecture of the segmentation-classification model. The model has three components: a double distillation fusion encoder, a multioutput separation decoder, and a fully connected classifier. The encoding part includes a 3D convolution layer and a pooling layer. The encoder has two different paths for extracting features from DW images and Tmax images. The decoding part generates two outputs: Mask (DWI) and Mask (Tmax). In the multioutput separation refinement module, the output is separated and refined step-by-step to obtain the final output result. Then, the fusion abstraction feature of the last layer of the encoder is used to generate binary classification results. This component includes a flattening operation, global average pooling, fully connected layers, and a sigmoid output function

**Fig. 3:**
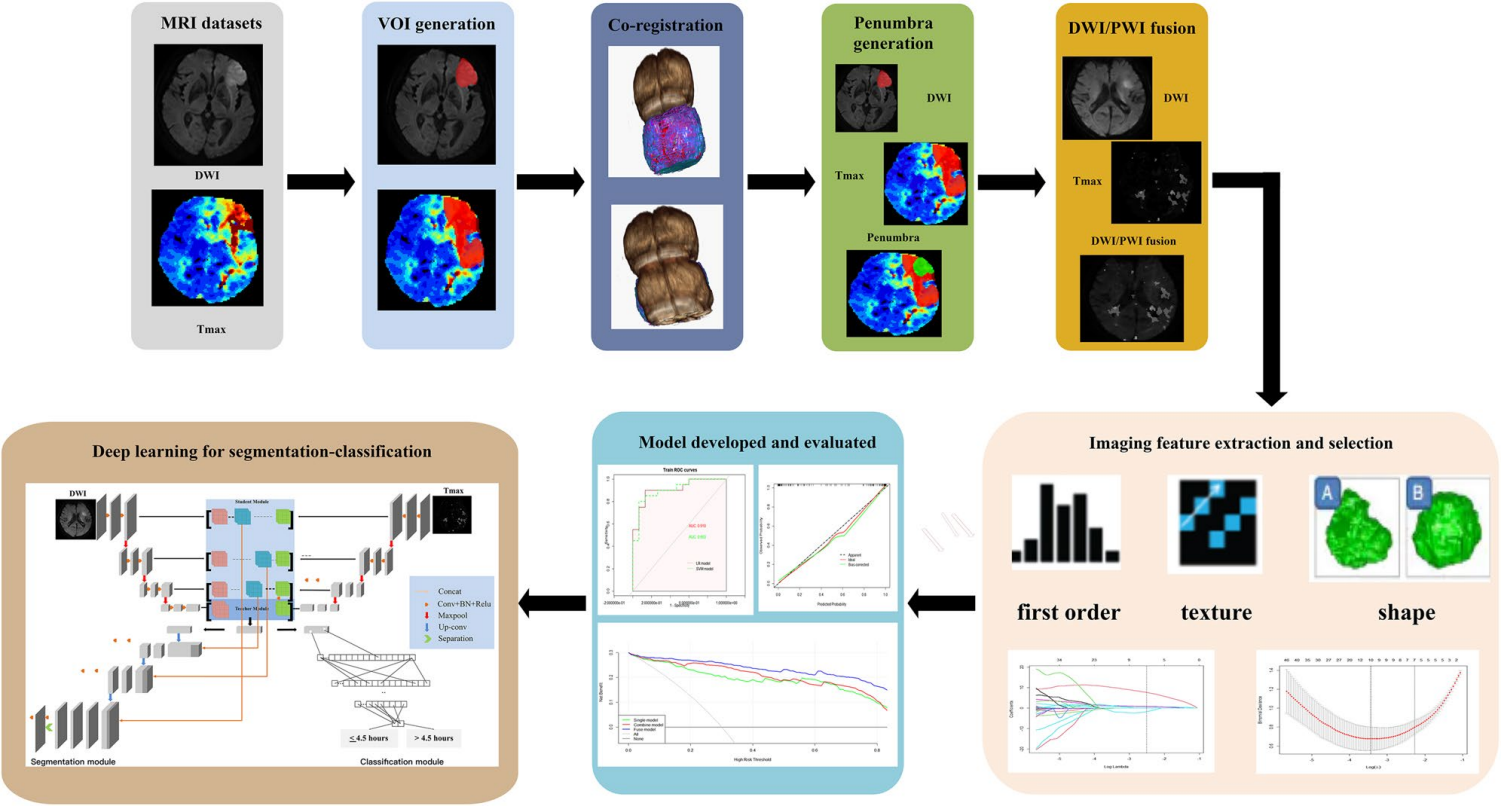
Classification and segmentation framework for identifying stroke onset time using DW and PW images proposed in this study

Segmentation and classification modules for identifying the onset time were designed in the same network frame, which we called X-Net, according to the shape of the model. The overall network architecture is shown in Fig. 2. The model consists of three components: a double distillation fusion encoder (differential distillation module and feature fusion module (Figure S1)), a multioutput separation decoding module (Figure S2), and a fully connected classifier. A detailed description of the model is provided in the Supplemental Materials (online). Additionally, conventional networks, including 2D Unet, 3D Unet, Vnet, and Attention Unet, were also used to segment DWI and Tmax images and compare them with X-Net. A few minutes are typically required to segment and classify a single patient with these methods. The classification and segmentation framework proposed in this study is shown in Fig. 3.

### Statistical analysis

Statistical analyses were performed using the statistical software R Studio (version 4.0.3). The Kolmogorov–Smirnov statistical test was used to test the normality of continuous variables. Continuous variables are presented as medians (interquartile ranges) and were assessed by Student’s *t* tests and Mann–Whitney *U* tests. Categorical variables are presented as percentages and were assessed by the χ^2^ test. Receiver operating characteristic (ROC) curve analysis, area under the curve (AUC), sensitivity, specificity, and accuracy were calculated using the pROC package to compare the efficacy of each model. Decision curve analysis (DCA) was conducted to assess the utility of each model. Calibration curves were used to evaluate whether the predicted probability of the classification model was close to the real probability. The Dice coefficient, Jaccard coefficient, average surface distance (ASD), and 95% Hausdorff (HD_95) metric were calculated to evaluate the segmentation efficacy of each model. Ablation experiments were conducted by removing one or more modules, including the differential distillation, feature fusion, and multioutput separation decoding modules, to compare the segmentation-classification efficacy of the X-Net. The test set was further divided into nine groups according to the onset time, namely, 0–1 h, 1–2 h, 3–4 h, 4–5 h, 5–6 h, 6–7 h, 7–8 h, and greater than 8 h, to assess classification efficacy for the subgroups. All statistical tests were two-sided, and *p* values of less than 0.05 were deemed to indicate statistical significance.

## Results

### Subject classification

Of the 418 patients in the final study cohort, 214 underwent MRI within 4.5 h of symptom onset, and 204 underwent MRI greater than 4.5 h after symptom onset. According to the TOAST classification, two hundred and twenty-nine (54.78%) patients had LAA, 86 (20.57%) had CE, 58 (13.88%) had SAO, 8 (1.91%) had OC, and 37 (8.85%) had UND. The age distribution of the study patients was between 51 and 83 years, and the mean age of the patients was 68.45 ± 12.56 years. The baseline characteristics were not significantly different between the training set and test sets (Table S1). There was no significant difference in the baseline characteristics between the onset time ≤ 4.5 h and > 4.5 h groups (Table S2).

### Feature selection

After LASSO screening, 12 features in the DWI dataset, 4 features in the Tmax dataset, 7 features in the penumbra dataset, 5 features in the DP fusion dataset, 17 features in the DWI + Tmax dataset, and 9 features in the DWI + penumbra dataset were selected. The tuning parameter and LASSO coefficient associated with the onset time are shown in Figure S3. The weight coefficients of the DWI + Tmax dataset features (− 1.3−0.20) were stronger than those in either the DWI dataset or Tmax dataset features (− 0.15−0.30), and the GLSZM feature had the highest weights (− 1.3). After the penumbra was added, the DWI + penumbra dataset features had higher weight coefficients (1.31−0.45), among which the GLCM feature had the highest weights (− 1.31). The DP fusion dataset features yielded the highest weights (− 1.32−7.1), among which the GLCM feature had the highest relative weights (7.1). The detailed features and weight coefficients are shown in Figure S4.

### Machine learning model

**Fig. 4:**
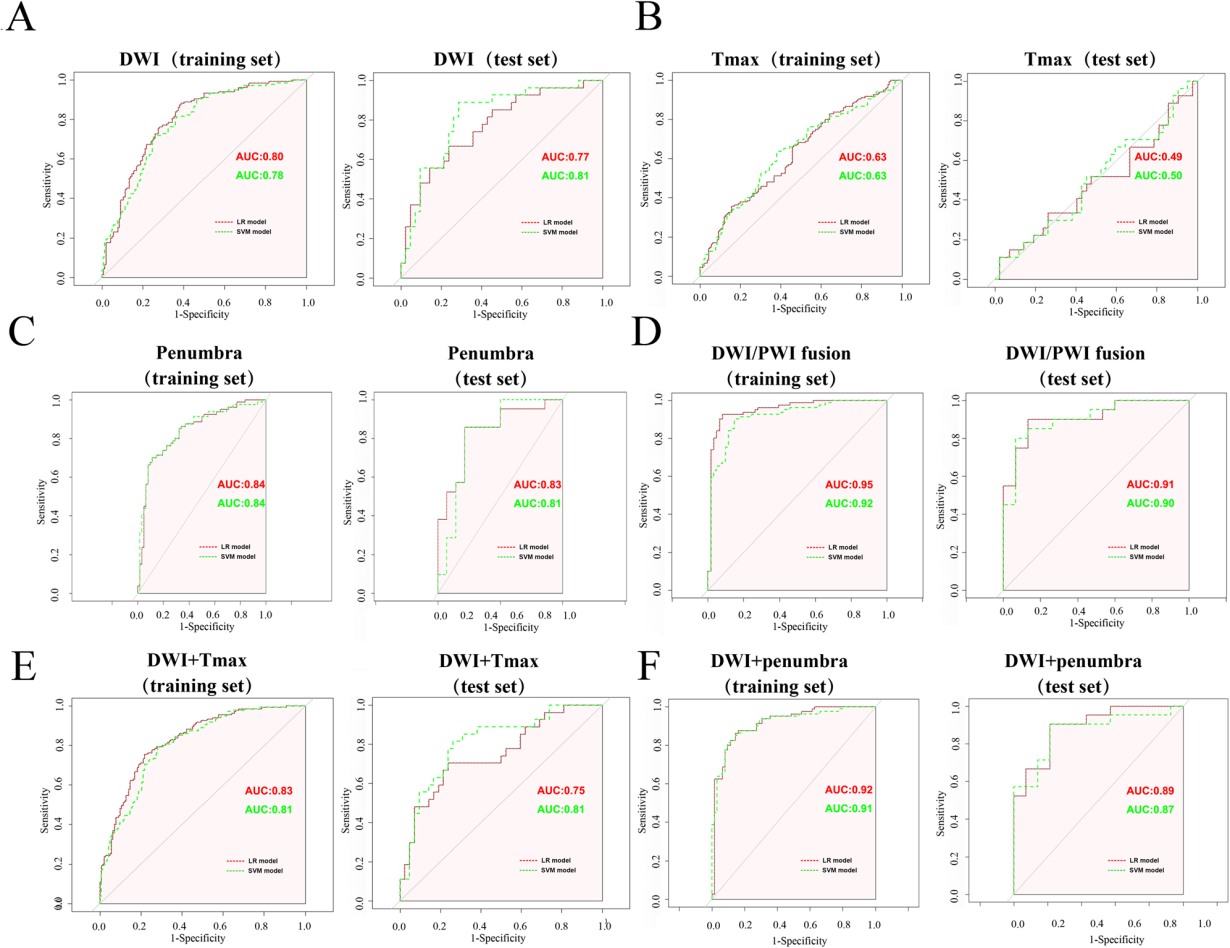
Receiver operating characteristic curves for identifying onset time in acute stroke patients based on DWI (**A**), Tmax (**B**), penumbra (**C**), DWI/PWI fusion (**D**), DWI+Tmax (**E**) and DWI+penumbra (**F**). The AUCs of models containing penumbra features were considerably better than those of the other models, and the DWI/PWI fusion model showed the highest AUC in identifying onset time in the training set (LR: 0.95; SVM: 0.92) and test set (LR: 0.91; SVM: 0.90). *AUC* area under the receiver operating characteristic curve, *LR* logistic regression, *SVM *support vector machine

The optimal LR and SVM models were assessed in their ability to identify each patient in the training and test sets. Figure 4 shows the ROC curves of the models in each dataset in identifying the onset time in acute stroke patients. The AUCs of the models based on the DWI + Tmax datasets (LR: 0.83 [95%CI: 0.60–0.99]; SVM: 0.81 [95%CI: 0.67–0.91]) were better than those of the models based on the DWI dataset (LR: 0.80 [95%CI: 0.67–0.91]; SVM: 0.78 [95%CI: 0.60–0.89]) or Tmax dataset (LR: 0.63 [95%CI: 0.45–0.92]; SVM: 0.63 [95%CI: 0.42–0.90]) in the training sets, but did not differ from those of models based on the DWI dataset in the test sets (DWI + Tmax model: LR: 0.75 [95%CI: 0.54–0.99], SVM: 0.80 [95%CI: 0.55–0.99]; DWI model: LR: 0.77 [95%CI: 0.42–0.99], SVM: 0.81 [95%CI: 0.52–0.99]). After the penumbra was added, the AUCs of the models were improved in both the training sets (penumbra model: LR: 0.84 [95%CI: 0.77–0.99], SVM: 0.84 [95%CI: 0.75–0.98]; DWI + Tmax model: LR: 0.92 [95%CI: 0.75–0.98], SVM: 0.91 [95%CI: 0.76–0.99]) and test sets (penumbra model: LR: 0.83 [95%CI: 0.61–0.99]; SVM: 0.81 [95%CI: 0.61–0.99]; DWI + penumbra model: LR: 0.89 [95%CI: 0.75–0.99], SVM: 0.87 [95%CI: 0.75–0.99]), and the DP fusion model showed the highest AUC in identifying the onset time in the training (LR: 0.95 [95%CI: 0.78–0.99]; SVM: 0.92 [95%CI: 0.77–0.99]) and test sets (LR: 0.91 [95%CI: 0.83–0.99]; SVM: 0.90 [95%CI: 0.80–0.99]). The sensitivity, specificity, and accuracy of the different models in the test set are shown in Table 1.

**Table 1:** Classification performance of machine learning models for the identification of patients within 4.5 h of symptom onset in test set

Classifier	Datasets	Accuracy	Sensitivity	Specificity	AUC
LR					
	DWI	0.70 (0.39–0.99)	0.71 (0.46–0.96)	0.54 (0.32–0.76)	0.77 (0.42–0.99)
	Tmax	0.52 (0.17–0.87)	0.54 (0.23–0.85)	0.44 (0.30–0.58)	0.49 (0.31–0.67)
	Penumbra	0.75 (0.59–0.91)	0.69 (0.57–0.81)	0.79 (0.61–0.97)	0.83 (0.61–0.99)
	DP fusion	0.81 (0.71–0.91)	0.79 (0.67–0.91)	0.90 (0.76–0.99)	0.91 (0.83–0.99)
	DWI + Tmax	0.68 (0.35–0.99)	0.71 (0.22–0.99)	0.60 (0.38–0.82)	0.75 (0.54–0.99)
	DWI + penumbra	0.80 (0.68–0.92)	0.77 (0.59–0.95)	0.88 (0.68–0.99)	0.89 (0.75–0.99)
SVM					
	DWI	0.71 (0.44–0.98)	0.72 (0.43–0.99)	0.56 (0.32–0.80)	0.81 (0.52–0.99)
	Tmax	0.55 (0.46–0.84)	0.55 (0.48–0.82)	0.47 (0.41–0.63)	0.50 (0.38–0.72)
	Penumbra	0.74 (0.60–0.88)	0.69 (0.55–0.83	0.78 (0.53–0.99)	0.81 (0.61–0.99)
	DP fusion	0.80 (0.72–0.88)	0.78 (0.66–0.90)	0.89 (0.73–0.99)	0.90 (0.80–0.99)
	DWI + Tmax	0.72 (0.55–0.99)	0.75 (0.40–0.99)	0.60 (0.49–0.91)	0.80 (0.55–0.99)
	DWI + penumbra	0.77 (0.65–0.89)	0.76 (0.56–0.96)	0.85 (0.69–0.99)	0.87 (0.75–0.99)

95% confidence interval is shown in parenthesis. *AUC* area under the receiver operating characteristic curve; *DWI* diffusion-weighted imaging; *DP* fusion diffusion–/perfusion-weighted imaging fusion; *LR* logistic regression; *SVM* support vector machine

### Clinical efficacy evaluation

**Fig. 5:**
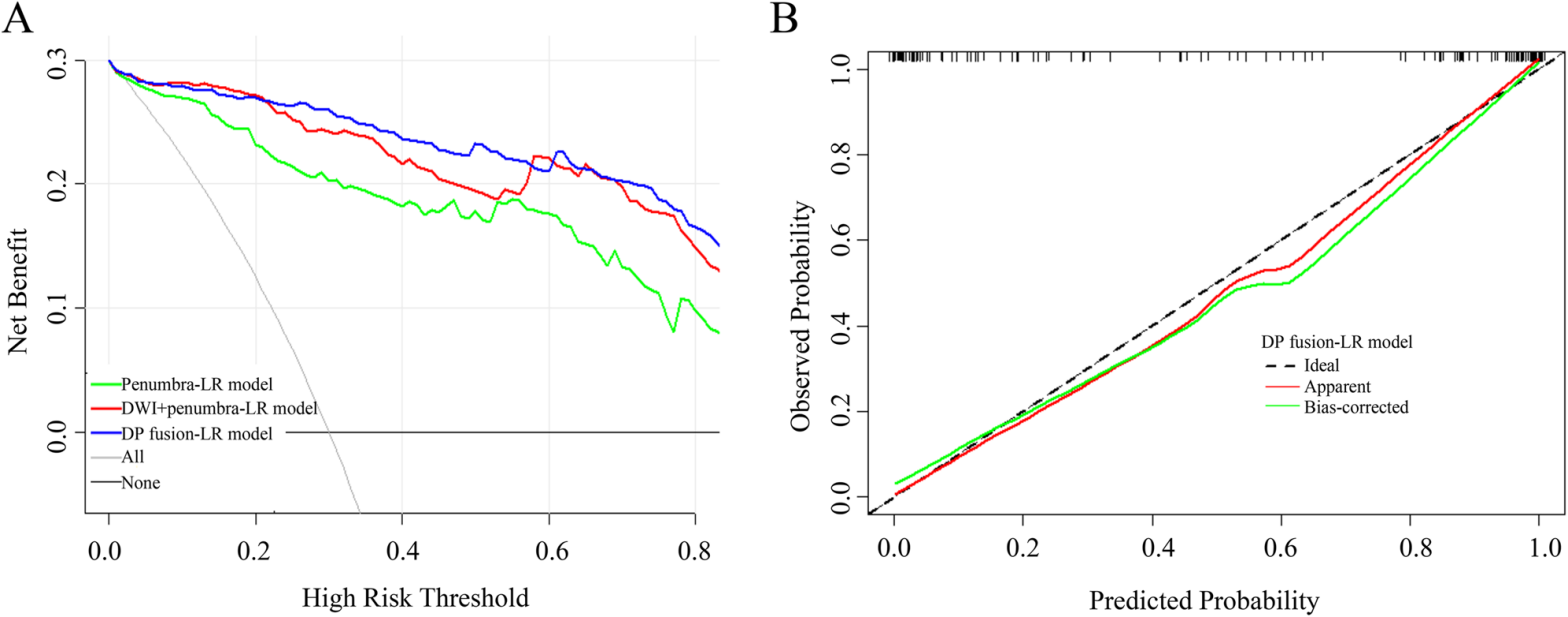
**A** Decision curve analysis for each model. The y-axis measures the net benefit, which is calculated by summing the benefits (true-positive findings). The decision curves show that the application of the DP fusion-based model for identifying acute stroke onset time was more beneficial than the addition of the penumbra or the DWI + penumbra model. **B** The calibration curve plot demonstrates the good calibration ability of the DP fusion-LR model, with an average absolute error of 0.049. DWI = diffusion-weighted imaging, DP fusion = diffusion/perfusion-weighted imaging fusion

Figure 5A demonstrates the decision curves of three models with good performance (penumbra-LR, DWI + penumbra-LR, and DP fusion-LR). Decision curve analysis graphically shows the clinical usefulness of a model based on the potential risk threshold (x-axis) and the net benefit of using the model to risk-stratify patients (y-axis) relative to the assumption that no patients will have an event (within 4.5 h). The DP fusion-LR model displayed consistent positive and greater net benefits across a broad range of risk thresholds than the penumbra-LR model and DWI + penumbra-LR model. The calibration curve demonstrated the good calibration ability of the DP fusion-LR model (average absolute error: 0.049) (Fig. 5B).

### Segmentation-classification performance

**Fig. 6:**
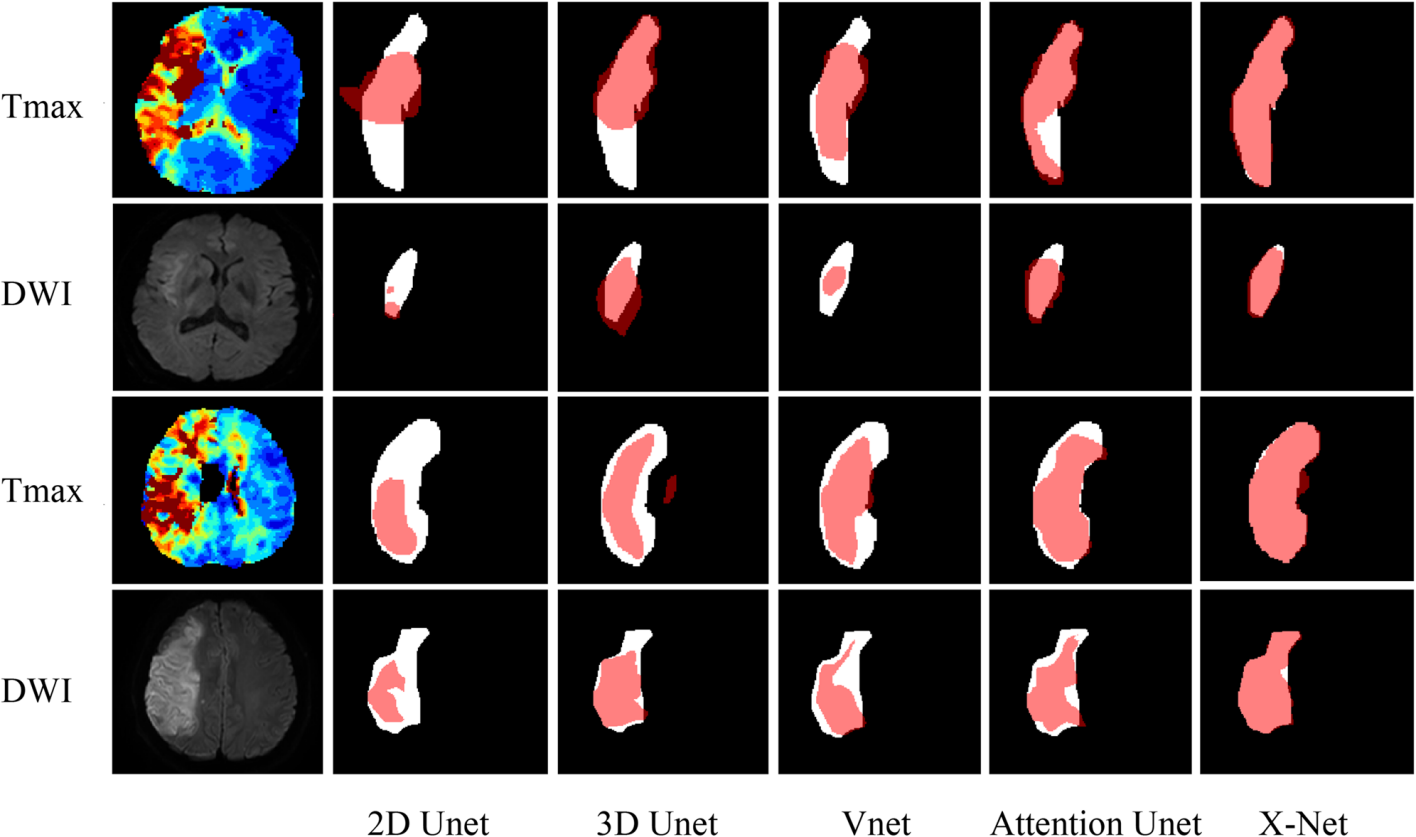
Visualization of the segmentation ischemic core in the DW images and the abnormal perfusion area in the Tmax images by using different networks, with the X-Net model showing the best performance. White regions represent the manually outlined VOIs, and red regions represent the automatically segmented VOIs. DWI = diffusion-weighted imaging, VOIs = volumes of interest

Table 2 demonstrates the segmentation performance of the different networks. Compared with the other four networks, the X-Net method obtained the highest Dice coefficient (DWI: 0.81; Tmax: 0.83) and achieved a performance close to that of the manual labeling method (AUC: 0.84 [95%CI: 0.70–0.98]). Some examples of DWI and Tmax segmentation using different networks are shown graphically in Fig. 6. In the ablation experiments, the segmentation-classification performance of the complete X-Net with the differential distillation, feature fusion, and multioutput separation decoding modules was significantly better than that of models built with individual or no modules (Table 3). In the subgroup analysis, model performance for the group of patients with an onset within 4–5 h of the MRI scan was lower than that for the other groups in all indicators, and the overall performance showed a weak linear relationship with time (Figure S5).

**Table 2:** Segmentation performance of different networks

Network	Images	Dice	Jaccard	ASD [voxel]	HD_95 [voxel]
2D Unet	DWI	0.68	0.59	2.82	1.52
	Tmax	0.75	0.62	8.37	7.68
3D Unet	DWI	0.74	0.62	2.73	1.02
	Tmax	0.81	0.70	4.12	1.45
Vnet	DWI	0.74	0.61	2.88	1.17
	Tmax	0.79	0.70	4.32	1.57
Attention Unet	DWI	0.76	0.64	2.74	0.95
	Tmax	0.81	0.70	4.27	1.44
X-Net	DWI	0.81	0.70	2.09	0.46
	Tmax	0.83	0.76	3.43	1.10

*ASD* average surface distance; *DWI* diffusion-weighted imaging; *HD_95* 95% Hausdorff

**Table 3:** Segmentation and classification performance of X-net networks with ablation experiments

Method	Images	Segmentation performance				Classification performance			
		Dice	Jaccard	ASD [voxel]	HD_95 [voxel]	Accuracy	Sensitivity	Specificity	AUC
X-Net	DWI	0.76	0.64	2.70	0.93	0.65 (0.49–0.81)	0.66 (0.50–0.82)	0.76 (0.68–0.84)	0.76 (0.66–0.86)
	Tmax	0.77	0.71	4.06	1.40				
X-Net with differential distillation module	DWI	0.78	0.66	2.63	0.86	0.69 (0.59–0.79)	0.69 (0.59–0.79)	0.78 (0.68–0.88)	0.78 (0.66–0.90)
	Tmax	0.82	0.74	3.83	1.36				
X-Net with feature fusion module	DWI	0.77	0.66	2.29	0.73	0.67 (0.59–0.75)	0.67 (0.59–0.75)	0.77 (0.67–0.87)	0.77 (0.65–0.89)
	Tmax	0.85	0.75	3.53	1.16				
X-Net with multioutput separation decoding module	DWI	0.80	0.68	2.21	0.56	0.66 (0.56–0.76)	0.68 (0.58–0.78)	0.79 (0.67–0.91)	0.81 (0.69–0.93)
	Tmax	0.81	0.74	3.54	1.19				
X-Net with three modules	DWI	0.81	0.70	2.09	0.46	0.75 (0.64–0.88)	0.72 (0.60–0.84)	0.83 (0.71–0.95)	0.84 (0.70–0.98)
	Tmax	0.83	0.76	3.43	1.10				

95% confidence interval is shown in parenthesis. *AUC* area under the receiver operating characteristic curve; *ASD* average surface distance; *HD_95* 95% Hausdorff

## Discussion

We developed and evaluated automatic machine learning models based on DWI and PWI images for identifying stroke within 4.5 h. The results showed that ML models based on DP fusion had the best performance (AUC ≥ 0.90) and the greatest net benefits among all models compared. To reduce the workload of the radiologist, we also proposed an automatic segmentation-classification method for identifying the onset time. The X-Net method proposed in our study achieved a performance similar to that of the manual labeling methods (AUC: 0.84).

A recent machine learning approach for identifying onset time using the DWI and PWI images of 131 acute stroke patients achieved an AUC of 0.77, with a sensitivity of 0.788 [13]. We achieved a better performance on a larger multicenter dataset, with an AUC reaching up to 0.91. The combination of DWI and PWI images has been shown to be beneficial for predicting onset time in acute stroke patients, and we observed similar results. In addition, after the penumbra features were introduced, the performance of the models was greatly improved. The average AUC in the test set was 0.81-0.91 for the model with the highest classification performance. Penumbra injury is reversible during the first few hours of ischemia, and the timely saving of the penumbra is critical to the outcome of acute stroke patients [23, 24]. According to our results, penumbra features can improve the prediction of onset time in acute stroke patients. In addition, the DP fusion models proposed in our study, built from features reflecting both texture and shading changes in the DWI and PWI images, achieved an AUC of 0.91, which is superior to the AUC of 0.765 reported by Ho et al [13], and the AUC of 0.851 reported by Lee et al [12], and the AUC of 0.840 obtained in our previous studies by using DWI and FLAIR images [14].

Although the DP fusion models had better performance in identifying the onset time than other ML models based on DWI and/or PWI, clinicians still need to delineate the VOIs on DWI and PWI. The heavy workload of manual labeling is not conducive to large-scale analyses, and penumbra information is critical in identifying onset time. In our study, a multitask learning method was used to unify the segmentation and classification in the same network, improving feature utilization and model accuracy. The X-Net model proposed in our study can realize both segmentation and classification tasks and achieve Dice coefficients of 0.81 on DWI images and 0.83 on Tmax images with an AUC of 0.84 in identifying onset time in the test set, much higher than that reported for DWI/PWI mismatch diagnosed by radiologists in the literature. Here, the lack of lesion edges and mildly high signals may be responsible for some false positives. In addition, the differential distillation module proposed in our study, borrowing ideas from knowledge transfer [25], was used to alleviate the local information difference caused by registration error, guiding local feature extraction using global abstract information. Furthermore, to prevent confusion in the output results of the two segmentation labels, a multioutput separation decoding module was added to separate and gradually refine the outputs before obtaining the final output results.

Ablation experiments are typically used for neural networks, especially for relatively complex neural networks. These experiments are used to help understand the performance of the network by deleting and studying parts of the network [26]. Our results showed that the segmentation-classification performance of the complete X-Net model with the differential distillation, feature fusion, and multioutput separation decoding modules was significantly better than that of models built with individual or no modules. When the above three modules were removed, the performance of the X-Net model decreased significantly. The classification and segmentation effects were improved when the differential distillation module was introduced. The model gradually transfers consistency differences from the highest level to the lowest level, alleviating local feature offsets caused by insufficient registration accuracy. Additionally, the extracted features are conducive to the task of identifying the stroke onset time. When the feature fusion module was added [27], the segmentation effect of the model was improved. Because both paths in the encoder have the same importance, the decoding part uses the two low-level details to ensure the accuracy of the DWI and Tmax segmentation results. Feature fusion can compress the features in each dimension. Thus, the overall performance of the features in this dimension is improved, and the model segmented images better. After the multioutput separation decoding module was added, the segmentation effect was considerably improved. The multioutput separation decoding module separates and gradually refines the outputs to obtain the final output result. By expanding and compressing the dimensionality of the feature map, the expression ability of the model is enhanced, and the segmentation effect is improved. Thus, the X-Net model proposed in our study has good segmentation-classification performance superior to that of other networks and can output two segmentation results with the same network, which reduces the time and difficulty of obtaining the results to a certain extent.

Our study had several limitations. First, the population in our study did not include all types of acute stroke patients. Because the image texture features and segmentation features cannot be reliably defined within small VOIs, patients with small infarctions (lesions < 1 cc in size) [12] were excluded from our study. Second, human reading results were not obtained. However, the results in our study are better than those obtained in our previous studies and other studies. Third, DWI or PWI sequence acquisition parameter variations may impact segmentation and classification performance. Therefore, the value of our approach needs to be further validated with multicenter data. Fourth, at present, there are many excellent convolutional neural networks, including UNet +  + (the improved version of UNet) [28, 29], VNet [30, 31], and ELNet [32], for extracting medical image data. The use of more advanced networks may further improve the training speed and the segmentation accuracy of the network. The structure of the proposed network could be further improved to obtain higher segmentation accuracy for lesions in acute stroke patients. The classification boundary of the binary classification task based on MR images is fuzzy. Classification problems with onset times greater or less than 4.5 h can be subdivided into multiclassification tasks to further evaluate the performance. Thus, the onset time classification label in acute stroke patients could be further refined.

## Conclusion

In conclusion, we developed an automatic machine learning model for identifying stroke within 4.5 h using images from two modalities (DWI and PWI). The DWI/PWI-based DP fusion model demonstrated the best performance in identifying the onset time among the evaluated models. To reduce the radiologist’s workload, we also proposed a segmentation-classification network (X-Net) and verified that it can achieve a performance close to that of the manual labeling method. We anticipate that this method could provide clinicians with decision-making guidance for acute stroke patients with unknown onset times.

## Supplementary Information

Below is the link to the electronic supplementary material.

Supplementary file1 (PDF 741 KB)

## Abbreviations

AUC: Area under the receiver operating characteristic curve
DP fusion: Diffusion/perfusion-weighted imaging fusion
DWI: Diffusion-weighted imaging
LASSO: Least absolute shrinkage and selection operator
LR: Logistic regression
PWI: Perfusion-weighted imaging
SVM: Support vector machine
VOI: Volume of interest

## Declarations

### Guarantor

The scientific guarantor of this publication is Xindao Yin.

### Statistics and biometry

No complex statistical methods were necessary for this study.

### Study subjects or cohorts overlap

No.

### Methodology

• retrospective

• randomized controlled trial

• performed at two institutions
