# Supplementary material for: Diffusion-/perfusion-weighted imaging fusion to automatically identify stroke within 4.5 h
Source: Eur Radiol. 2024 Mar 15;34(10):6808–19. doi: 10.1007/s00330-024-10619-5 (PMC11399198; doi:10.1007/s00330-024-10619-5)
Supplement: Supplementary file 1 — Supplementary file1 (PDF 741 KB) [file 330_2024_10619_MOESM1_ESM.pdf]

Diffusion/Perfusion-Weighted Imaging Fusion to Automatically Identify Stroke within 4.5 Hours

Electronic Supplementary Material (ESM)

Eur Radiol (2024) Jiang L, Sun J, Wang Y et al.

Contents

Supplemental Methods

MRI Protocol and Processing

Deep Learning for Lesion Segmentation and Classification

Table S1. Baseline Characteristics of the Study Population

Table S2. Baseline Characteristics Comparison between Onset Time ≤ 4.5 Hours and >4.5 Hours

Figure S1. Schematic of the feature fusion module.

Figure S2. Schematic of the multioutput separation decoding module.

Figure S3. Radiomic feature selection using LASSO logistic regression and LASSO coefficient profiles of the radiomic features.

Figure S4. The detailed features associated with acute stroke onset time and the weight coefficients of different datasets after LASSO screening.

Figure S5. Line graphs of performance in different onset time subgroups.

Supplemental Methods

MRI Protocol and Processing

The MRI protocol included DWI (spin-echo (SE) sequence; repetition time (TR), 2501 ms; echo time (TE), 98 ms; acquisition matrix, 152 × 122; 3 directions; field of view (FOV), 230 × 230 mm; flip angle (FA), 90°; slices, 18; slice thickness, 6 mm; intersection gap, 1.3 mm; b values, 0 and 1000 s/mm^2; and scan time, 30 s) and PWI (T2*-weighted gradient recalled echo (T2*GRE) sequence; TR, 2000 ms; TE, 30 ms; acquisition matrix, 96 × 93; FOV, 224 × 224 mm; FA, 90°; slice thickness, 4 mm; and scan time, 88 s). Fifty phases and 20 images were obtained from each phase. During dynamic acquisition, a dose of 0.1 mmol/kg contrast agent (Magnevist, Bayer Schering Pharma) was injected at a rate of 4 mL/s.

Deep Learning for Lesion Segmentation and Classification

Double distillation fusion encoder: The encoder included two different feature extraction paths for the DWI images and Tmax images. The encoding part was composed of a 3D convolution layer and a pooling layer. To alleviate the local information difference caused by registration error and use global abstract information to guide local feature extraction, a difference distillation module based on the idea of knowledge transfer was proposed in this study. The differences between the global abstract features in the DWI and Tmax images were taken as Teacher, and the differences between other local features were taken as Student. The global difference information was transferred to the local area to ensure the information was as close as possible to the global difference.

Differential Distillation Module: The last layer of the encoding network was used as Teacher and the difference between the outputs of the DWI and Tmax paths was "taught" to the previous layer in the same model. Suppose that the output feature maps of two adjacent convolution blocks are (F_{DWI}^{m}, F_{Tmax}^{m}) and (F_{DWI}^{m+1}, F_{Tmax}^{m+1}); then, the consistency difference is shown in the following equation:

$$D_m = \sum_{c=1}^{N_m} |F_{DWI}^{m}[c,:,:,:] - F_{Tmax}^{m}[c,:,:,:]|$$

In the formula, the subtraction operation is performed pixel-by-pixel, c is a channel in the feature map, and $\mathrm{N_m}$ is the number of channels in layer M of the feature map. Thus, according to the design of the loss function, the output consistency of the earlier network layer tends to approach that of the later layer. The loss function is shown in the following equation:

$$L_{distil} = \sum_{m=1}^{M-1} ||S(I(D_m)) - S(D_{m+1})||^2$$

where, M is the level of the encoding network, and I(.) is a trilinear interpolation operation that ensures the 3D feature maps have the same scale, and S(.) is the softmax operation, which is used to normalize consistency differences to between [0, 1].

Feature Fusion Module: In this task, the two paths of the encoding part had

the same importance, and the decoding part needed to use the two low-level details to ensure the accuracy of the DWI and Tmax segmentation results. Suppose that the output feature maps of a layer in the encoder are $F_{DWI}(\mathrm{C,D,H,W})$ and $F_{Tmax}(\mathrm{C,D,H,W})$. Then, the features of a dimension can be compressed to obtain the overall performance of the features in this dimension:

$\phi^{Depth}(F_{DWI}) = \sum_{i=1}^{D} d_i F_{DWI}[:,i,:,:], \phi^{Depth}(F_{Tmax}) = \sum_{i=1}^{D} d_i F_{Tmax}[:,i,:,:]$

$\phi^{Height}(F_{DWI}) = \sum_{j=1}^{H} h_i F_{DWI}[:,:,j,:], \phi^{Height}(F_{Tmax}) = \sum_{j=1}^{H} h_i F_{Tmax}[:,:,j,:]$

$\phi^{Width}(F_{DWI}) = \sum_{k=1}^{W} w_i F_{DWI}[:,:,:,k], \phi^{Width}(F_{Tmax}) = \sum_{k=1}^{W} w_i F_{Tmax}[:,:,:,k]$

where, d_i, $h_i$and w_i are the weights of different layers in each dimension, which are learnable parameters. Therefore, the fusion features in each dimension are as follows:

$$F_{fuse}^{D} = \phi^{Depth}(F_{DWI}) + \phi^{Depth}(F_{Tmax})$$

$$F_{fuse}^{H} = \phi^{Heigh}(F_{DWI}) + \phi^{Heigh}(F_{Tmax})$$

$$F_{fuse}^{W} = \phi^{Width}(F_{DWI}) + \phi^{Width}(F_{Tmax})$$

Then, the final fusion feature F_{fuse} is obtained by adding and combining the features of all dimensions and refining them through a 1×1×1 convolution:

$$F_{fuse} = Conv_{1*1*1}(\mathrm{B}^{D}(F_{fuse}^{D}) + \mathrm{B}^{H}(F_{fuse}^{H}) + \mathrm{B}^{W}(F_{fuse}^{W}))$$

where, B^{D}, B^{H} and B^{W} are broadcast operations in each dimension that restore the compressed feature maps to their initial dimensions. Fig S1 shows the overall process.

Multioutput Separation Decoding Module: The decoding part generated two types of outputs: Mask (DWI) and Mask (Tmax). In this paper, a multioutput separation refinement module was constructed, and the output was separated and refined step by step to obtain the final output result. As shown in Fig S2, after the model finally produced feature maps with the number of N voxels, the number of feature maps was doubled through a 1×1×1 convolution, and the feature graphs were divided into 2 groups according to their sequences, with N

feature graphs in each group. Conv (3×3×1) was used to ensure that the two groups of features obtained by sequential splitting corresponded to two different masks. Then, the dimension of the feature map was expanded and compressed to refine the mask.

Fully Connected Classifier: The fusion abstraction features of the last layer in the encoder were used to generate the binary classification results. This component was mainly composed of the flatten operation, global average pooling, fully connected layers and the sigmoid function. The flatten operation flattened the high-dimensional feature map into a one-dimensional vector, and global average pooling was used to reduce the calculations of full connections. The sigmoid function was used to generate a classification probability value in the range [0,1], and the final classification result of the onset time was obtained after setting a certain threshold value.

The foreground (lesion area) varied with the onset time for different patients, and the foreground scale varied greatly; thus, the cross- entropy cannot be used as a loss function alone. Therefore, we combined the cross-entropy and Tversky losses to design the loss function. The binary cross-entropy loss function is shown in the following equation:

$$Loss_{CE} = -\frac{1}{N}\sum_{n=1}^{N}(g_n \log(p_n) + (1 - g_n)\log(1 - p_n))$$

where N is the number of voxels in the image, g_n is the actual classification of the nth voxel in the image to be divided as the ground truth, and p_n is the probability value predicted according to the position of the voxel. The definition of Tversky loss function is shown in the following equation:

$$Loss_{Tversky} = \frac{\sum_{n=1}^{N} p_n g_n + \epsilon}{\sum_{n=1}^{N} p_n g_n + \alpha(\sum_{n=1}^{N} p_n(1 - g_n)) + \beta(\sum_{n=1}^{N}(1 - p_n)g_n) + \epsilon}$$

where ϵ is a smoothing parameter that prevents the numerator and denominator from being 0, $p_n(1 - g_n)$ represents false positives, and $(1 - p_n)g_n$ represents false negatives. The perfusion ischemia region (Tmax)

of a patient is generally large, and there are no serious category imbalances; thus, α and β are 0.5 and 0.5, respectively. However, because the core infarct region of a patient progresses continuously according to the onset time, there may be a serious category imbalance in the early stage of onset. Therefore, the model paid more attention to the segmentation of the foreground region, and α and β were 0.3 and 0.7, respectively. In summary, the total loss of the segmented part is shown in the following formula:

$$Loss_{Seg} = Loss_{CE}^{Dwi} + Loss_{Tversky(0.3,0.7)}^{Dwi} + Loss_{CE}^{Tmax} + Loss_{Tversky(0.5,0.5)}^{Tmax}$$

The output of the X-Net classification component was the predicted category probability, and binary classification results can be generated through threshold setting. In this component, the cross-entropy was used as the loss function, as shown in the following equation:

$$Loss_{Cls} = -(y_n * \log(z_n) + (1 - y_n) * \log(1 - z_n))$$

where z_n represents the probability that the onset time of the nth patient predicted by the model was more than 4.5 hours, and y_n represents the actual onset time label of the patient. The loss function of the fully supervised component of the model proposed this paper is shown in the following equation:

$$Loss_{Super} = \gamma_1 Loss_{Seg} + \gamma_2 Loss_{Cls} + \gamma_3 L_{distil}$$

where, γ_1, γ_2 and γ_3 are the weight coefficients that balance the three kinds of losses.

Table S1. Baseline Characteristics Comparison between Training Set and Test Set

Metric	Training Set (n=335)	Test Set (n=83)	*P*
**Age, median (IQR)**	68 (61, 78)	71 (63, 80)	0.126
**Male, n (%)**	213 (63.6)	51 (61.4)	0.815
**NIHSS on admission, median (IQR)**	9 (5, 14)	9 (5, 14)	0.748
**Time from onset to MRI (min), median (IQR)**	270 (210, 450)	270 (180, 390)	0.361
**Onset time within 4.5 hours, n (%)**	171 (51.0)	43 (51.9)	0.901

Abbreviations: Numbers are n (%) or median (interquartile ranges), as appropriate. NIHSS National Institutes of Health Stroke Scale; MRI magnetic resonance imaging.

Table S2. Baseline Characteristics Comparison between Onset Time ≤4.5 Hours and >4.5 Hours

Metric	Training Set			Test Set		
	onset time ≤4.5 hours (n=171)	Onset time >4.5 hours (n=164)	*P*	onset time ≤4.5 hours (n=43)	onset time >4.5 hours (n=40)	*P*
**Age, median (IQR)**	66 (60, 76)	68 (61, 79)	0.114	70 (63, 81)	69 (62, 79)	0.132
**Male, n (%)**	103 (60.2)	110 (67.1)	0.193	26 (60.5)	25 (62.5)	0.849
**NIHSS on admission, median (IQR)**	10 (4, 14)	9 (5, 16)	0.532	10 (5, 13)	8 (5, 13)	0.813
**Time from onset to MRI (min), median (IQR)**	265 (200, 450)	270 (220-450)	0.134	260 (190, 400)	270 (180, 410)	0.238

Abbreviations: Numbers are n (%) or median (interquartile ranges), as appropriate. NIHSS National Institutes of Health Stroke Scale; MRI magnetic resonance imaging.

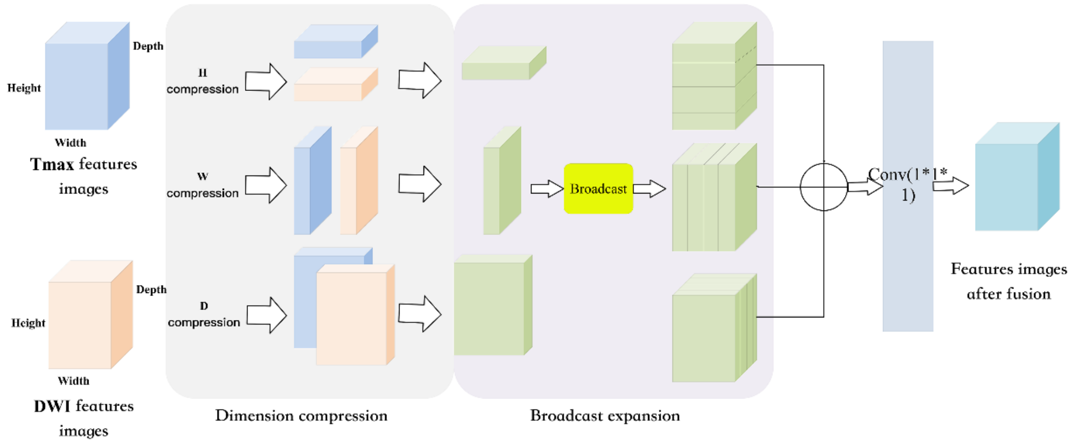

Figure S1. Schematic of the feature fusion module. The two paths in the encoding part had the same importance, and two low-level details were used in the decoding part to ensure the accuracy of the DWI and Tmax segmentation results.

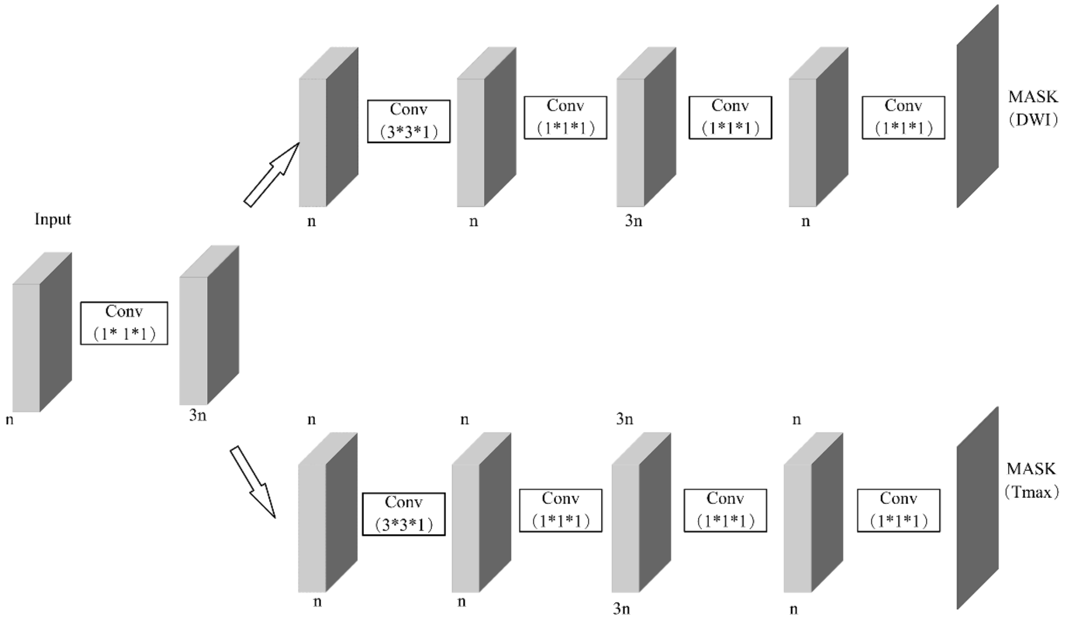

Figure S2. Schematic of the multioutput separation decoding module. After the model produced feature maps N voxels, the number of feature maps was doubled through 1×1×1 convolutions, and the feature graphs were divided into 2 groups according to their sequences, with N feature graphs in each group. Conv (3×3×1) was used to ensure that the two groups of features obtained by sequential splitting corresponded to two different masks. Then, the dimension of the feature map was expanded and compressed to refine the mask.

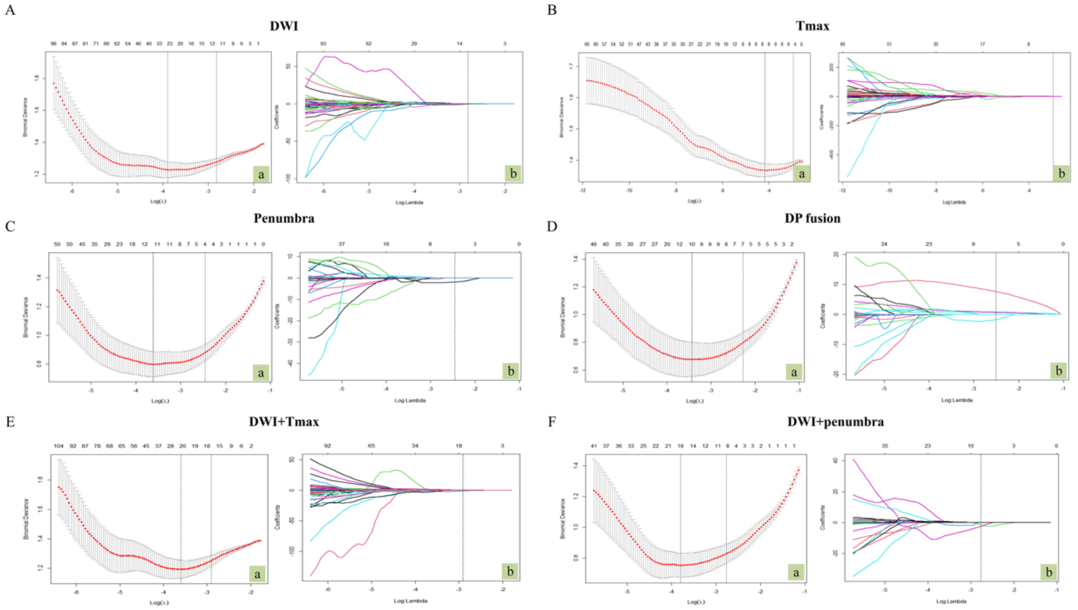

Figure S3. Radiomic feature selection using LASSO logistic regression and LASSO coefficient profiles of the radiomic features. a. The tuning parameters in the least absolute shrinkage and selection operator model were selected using 10-fold cross-validation via minimum criteria. The area under the curve was plotted vs. log (λ). Dotted vertical lines were drawn at the optimal values according to the minimum criteria and the 1 standard error of the minimum criteria (the 1 - standard error criteria).

b. A vertical line was plotted at the optimal λ value. LASSO = least absolute shrinkage and selection operator.

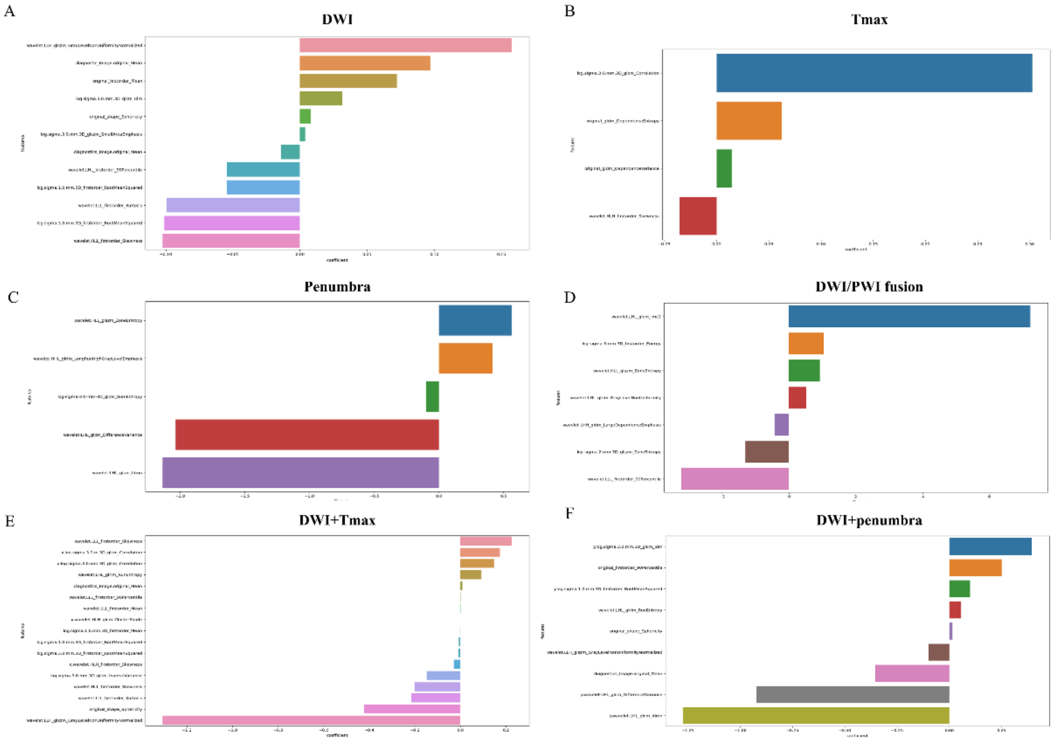

Figure S4. The detailed features associated with acute stroke onset time and the weight coefficients of different datasets after LASSO screening. The weight coefficients of the DWI+Tmax dataset (-1.3~0.20) were stronger than those of either the DWI dataset or the Tmax dataset (-0.15~0.30). After the penumbra was added, the weight coefficients of the DWI+penumbra dataset were slightly higher, with values of 1.31~0.45, and the weight coefficients of the DP fusion dataset were significantly higher, with values of -1.32~7.1. DWI = diffusion-weighted imaging, DP fusion = diffusion/perfusion- weighted imaging fusion, LASSO = least absolute shrinkage and selection operator.

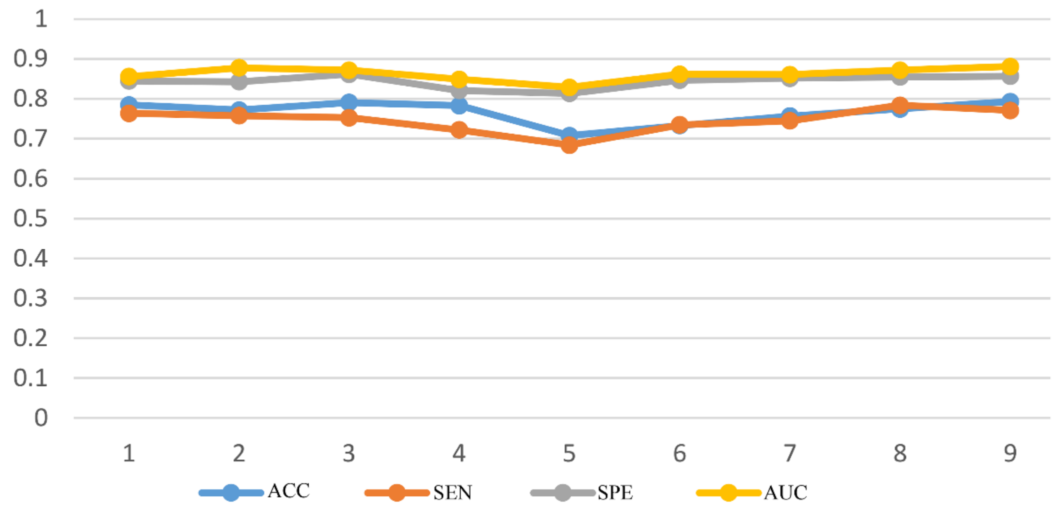

Figure S5. Line graphs of performance in different onset time subgroups. The group of patients within 4-5 hours was lower than the other groups in all indicators, and the overall performance showed a weak linear relationship with time.
